# Volunteer Engagement within Equine Assisted Services

**DOI:** 10.3390/ani14020249

**Published:** 2024-01-12

**Authors:** Aviva Vincent, Meghan Morrissey, Mary Acri, Fei Guo, Kimberly Hoagwood

**Affiliations:** 1 School of Social Work, College of Health, Cleveland State University, Cleveland, OH 44115, USA; 2 Langone Health, New York University, New York City, NY 10016, USA; megamorrissey@gmail.com (M.M.); mary.acri@gmail.com (M.A.); kimberly.hoagwood@nyulangone.org (K.H.); 3 Department of Child and Adolescent Psychiatry, Grossman School of Medicine, New York University, New York City, NY 10016, USA; fei.guo@nyulangone.org; 4 Department of Population Health, Grossman School of Medicine, New York University, New York City, NY 10016, USA

**Keywords:** volunteer, equine assisted services, oxytocin, cortisol, adaptive riding, children and youth, anxiety, horses

## Abstract

**Simple Summary:**

Volunteering is an important experience to millions of Americans, and moreover, it is imperative to the sustainability of the organizations they give time to. This study considers the effect of volunteering within Equine Assisted Services for adaptive horseback riding lessons. Volunteers directly impact the rider-horse bond by increasing accessibility to horseback riding for individuals with disabilities, fostering a meaningful bond between the rider and horse. As part of a larger study entitled *Reining in Anxiety*, the researchers collected physiological data (e.g., pooled saliva) and survey responses from volunteers (*n* = 41) to explore volunteer’s experiences. The data from the saliva analysis and surveys provided insight into experiences of stress and affiliative bonding with the riders and horses. The results also explored satisfaction with volunteering. There was a non-significant, positive trend in oxytocin and alpha-amylase, while cortisol remained level. The responses in the survey suggested that volunteers perceive their role positively, as they shared nuanced experiences of responsibility to ensure safety, and enjoyment in assisting the riders. The complex emotions and experiences of volunteers are important to understand to create meaningful, sustainable volunteer engagement. This is particularly important in the EAS industry, where in volunteers are vital to the safety of the rider, horse, and the industry, which is reliant on volunteerism.

**Abstract:**

This study examines the effect of volunteering within a Professional Association of Therapeutic Horsemanship International (PATH Intl) premiere accredited center by exploring the experiences of volunteers leading horses in adaptive riding lessons. Adaptive Riding lessons are horseback riding lessons for individuals ages four through the lifespan, with special needs, varying from cognitive, physical, social-emotional, or other challenges. Volunteers directly impact the rider-horse bond by increasing accessibility to horseback riding for individuals with disabilities, fostering a meaningful bond between the rider and horse. The research questions were as follows: (1) do saliva measures of cortisol and alpha-amylase (stress), and oxytocin (affiliative bonding) change over time for volunteers; and (2) how satisfied are volunteers with volunteering for Equine Assisted Services (EAS)? Forty-one volunteers participated in *Reining in Anxiety*, an intervention combining adaptive riding and cognitive behavioral therapy. Physiological data (i.e., pooled saliva, saliva combined from various glands throughout the mouth, resting under the tongue prior to collection) were collected pre/post riding session at four time points during the 10-session intervention, measuring oxytocin, cortisol, and alpha-amylase. Post-intervention, volunteers completed a survey about their experiences as volunteers and as participants in the study. All saliva samples were collected successfully. There was a non-significant, positive trend in oxytocin and alpha-amylase, while cortisol remained level. The responses in the survey suggested that volunteers perceive their role positively, with nuanced experiences of a sense of responsibility to ensure safety, and enjoyment in assisting the riders. Volunteers are vital to the safety of the rider and horse. While their perceived and internalized responsibility is evidenced by an increase in stress (e.g., cortisol remaining level and an increase in alpha-amylase), it is not necessarily negative stress, as there is simultaneously affiliative bonding expressed (oxytocin). The complex emotions and experiences of volunteers are important to understand to create meaningful, sustainable volunteer engagement. This is particularly important in the EAS industry, which is reliant on volunteerism.

## 1. Introduction

Over 60 million Americans, or three out of ten, volunteer annually, which equates to 4.1 billion service hours and is worth 122.9 billion dollars [1]. Volunteers’ time is valued at $31.80 per hour [2]. 

The EAS industry is reliant on volunteers to meet the demand of serving participants, and the industry simply could not afford to hire the people needed to operate successfully and safely. Volunteers directly impact the rider-horse bond by increasing accessibility to horseback riding for individuals with disabilities; without volunteers serving as leaders of horses, many participants would not be able to safely ride. Furthermore, the relationship between the rider and volunteer, as well as the volunteer and horse is a meaningful bond that drives commitment to service. The EAS industry rests on the commitment of volunteers [3]. According to the Professional Association of Therapeutic Horsemanship International (PATH Intl), as of 2022, 49,705 individuals volunteered across 813 therapeutic riding centers to serve over 53,399 individuals. The same 813 centers are staffed by 5424 certified professionals, indicating that there were 44,281 more volunteers than professionals in 2022 [3]. While PATH Intl has the most robust statistics of the EAS industry, there are EAS facilities that are not PATH Intl members, and thus not represented in the data. 

An environmental scan of the literature found 98 articles of comparable content, though. Only five of the articles [4,5,6,7,8] specifically referenced EAS program volunteers as the participants of an empirical study. One article specifically focused on volunteers in youth-serving organizations with animals [4], whereas Culp and Bullock [5] studied volunteer outcomes from a 4-H training program. Only one study focused on the experiences of volunteers [6]. Rudd and colleagues’ [7] study found that who received training in equine health was significantly more accurate in identifying equine behavior. There is no empirical investigation into stress or satisfaction experienced by volunteers during adaptive riding lessons.

Despite the value of volunteers, exclusion in research has created significant gaps in knowledge. To date, this study is only the sixth to focus on volunteers, and the first to use mixed-methods to explore the volunteer’s experience in EAS. As research begins to intentionally include volunteers of EAS, their impact on the horse can also be strategically explored. A greater understanding of the volunteer’s perceptions and experiences will impact knowledge about the volunteer’s impact on the horse, and thereby support equine-welfare practices. An indirect outcome may be establishing evidence of the positive benefits of volunteering in EAS that further promote the sustainability of volunteerism and equine-welfare. The theoretical framework applied are psychological contract theory, the relationship between an organization and volunteer [9], and the “Social Cure”, which suggests positive effects from participating in a group especially if doing so includes personal growth, social connectedness, self-rated health, and well-being [4,5,6]. 

This study was undertaken to contribute knowledge to the literature about the physiological effects and perceived benefits of volunteering [10,11,12]. This study extends the current evidence base by triangulating self-reports with an analysis of saliva to measure cortisol and alpha-amylase (stress indicators), and oxytocin (affiliative bonding, defined as “valuing and enjoying interpersonal closeness with others” [13]). Given the current gaps in knowledge and the limitations of current research, this study was undertaken in order to contribute knowledge about the physiological effects and perceived benefits of volunteering to a research study that tested Reining in Anxiety (RiA), a 10-week manualized intervention that combines adaptive riding and core concepts of cognitive behavioral therapy [14,15,16]. 

Cortisol and alpha-amylase provide uniquely different interpretations of a person’s experience in an environment. Cortisol is often an indicator of prolonged stress in the body’s hypothalamic-pituitary-adrenal axis, while alpha-amylase is a surrogate indicator of acute stress and is experienced in the body’s autonomic nervous system. Thus, stress experienced by volunteers may be emotionally negative and/or representative of an increase in physical or mental effort [14]; due to this, both analytes are required to explore this complexity. Stress may be psychological, emotional, or physiological, thus, the indicators aid understanding of nuanced interpretation of stress [15,16,17]. 

Applied to this study, stress experienced by volunteers may be emotionally negative and/or representative of an increase in effort (i.e., physical effort, mental effort) [18]; due to this, both analytes are required to explore this complexity. Stress may be psychological, emotional, or physiological, thus, taken together, the indicators help researchers understand the nuanced interpretation of physiological stress [16,18,19,20]. This is the first known study to track physiological changes among volunteers in an adaptive riding program. Tracking physiological changes is important in combination with survey data to explore volunteers’ congruence of experience. The research questions that guided the exploration of volunteerism were as follows: (1) do saliva measures of cortisol and alpha-amylase (stress indicators), and oxytocin (affiliative bonding) change over time for volunteers; and (2) how satisfied are volunteers with volunteering for an EAS? 

## 2. Materials and Methods

### 2.1. Setting

This study was part of a larger randomized controlled trial that tested the effectiveness of a novel adaptive program entitled *Reining in Anxiety* (RiA), which is a therapeutic program for youth with mild-to-moderate anxiety delivered in an adaptive riding setting by Certified Therapeutic Riding Instructors (CTRI). This study was approved by the Biological Research Alliance of New York (BRANY) Institutional Review Board (20-10-604-826). The research took place at Fieldstone Farm Therapeutic Riding Center (FSF), a premiere Professional Association of Therapeutic Horsemanship International (PATH)-accredited facility in Northeast Ohio. The Center is located on 45 acres, with 40 horses and 19 CTRIs. A CTRI is a credentialed professional through PATH International, who provide mounted or unmounted adaptive/therapeutic horseback riding lessons to individuals with special needs. Adaptive horseback riding lessons are often supported by 1–3 volunteers per rider, one who physically leads the horse until the rider can do so independently, and one or two to sidewalk (to walk next to the person riding the horse for the duration of their lesson with significant, minimal, or no physical contact/support as directed by the Certified Therapeutic Riding Instructor and rider) for physical, emotional, or cognitive support. 

### 2.2. Intervention

**Figure 1:**
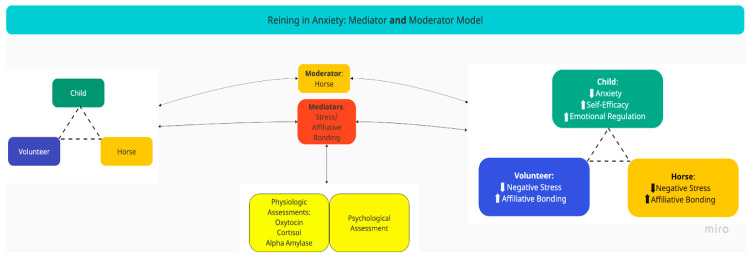
Conceptual Model of *Reining in Anxiety*.

RiA consists of 10 weekly sessions in a group format. Each session is approximately 45-min in length and includes both mounted and unmounted equine interaction activities to develop horsemanship skills, but also adds an intentional focus on mental health goals. Each session focuses on skill development of a specific component of cognitive behavioral therapy (CBT, e.g., restructuring negative thoughts) using repetition, and is reinforced through a weekly homework journal to promote generalization of skills outside of sessions. Sessions also included ample opportunity for horsemanship skill-building adaptable to the rider’s current skill level. The volunteers served as leaders for each of the riders throughout the intervention. The caregiver psychoeducation component was delivered in 7-min segments at the end of each session, with the intention to relay information to the caregiver, as well as an opportunity for instructors to assess rider skill knowledge retention. All learning was supported by both a website and written psychoeducation for caregivers, see references [10,11,12]. Figure 1 offers a conceptual model of the intervention. 

### 2.3. Procedure

In 2021, volunteers who participated in the larger randomized controlled trial were approached by a member of the research team about participating in the current study (IRB approval from BRANY # 23-12-178-826). If interested, the volunteers met with the researcher to learn about the RiA intervention and their role as a volunteer. If they expressed a desire to participate, they then consented to the study. Inclusion criteria included the following: current volunteer, commitment to the 10-week session, and a willingness to provide a saliva sample and complete measures as requested throughout the study. All 41 approached volunteers consented to participate in the study, which consisted of (1) providing 1 mL of pooled saliva before and after four sessions of the intervention and (2) completing a brief online satisfaction survey at the end of the study. 

### 2.4. Data Collection

Sociodemographic characteristics were captured by the Center as typical protocol. As part of the consent process, volunteers were made aware that the research team would access their name, basic demographic information (age, gender, and zip code of residence), volunteer roles, and schedule for volunteering. 

Oxytocin, cortisol, and alpha amylase saliva samples were collected prior to starting and ending the scheduled adaptive riding lesson in weeks one, four, seven, and ten of the intervention. To collect saliva, volunteers were asked to avoid food up to one hour before volunteering and to minimize drinking to ensure samples were not contaminated. Since collection occurred while COVID-19 precautions were in place, volunteers typically relocated to the stable aisles (outdoor space) to unmask and provide their sample. All health guidelines (i.e., sterile gloves, hand sanitizer, masking) were upheld throughout the study. Volunteers provided 1 mL of pooled saliva into a labeled vial with the volunteer’s unique identification number directly before and after volunteering. The vials were immediately placed in the freezer. All samples were stored in a secure, locked freezer. After week ten of the intervention, all samples were shipped on dry ice to Salimetrics LLC. (Carlsbad, CA, USA) for appropriate assays [15]. A log of sample times was maintained. Raw data were returned to the research team for analysis. 

Satisfaction with volunteering was collected via a research-informed survey administered after the 10-week program. The survey questions asked respondents for their opinion about the following: the research process, perception of safety, thoughts about the therapeutic benefit of riding, and questions about why they volunteer. Two question sets used three-point a Likert scale with a response set ranging from “Strongly Disagree/1” to “Strongly Agree/3”:

- When thinking about the research process, please rate how much you agree with the following statements: I understood why the spit samples were being collected; It was burdensome (time) to provide the spit samples; It was burdensome (process of spitting) to provide the spit samples.
- When thinking about the research process, please rate how much you agree with the following statements: It was burdensome to be part of the research study; I was excited to be a part of the research study; I was interested to learn about the results of the research study.

One question set used a range of “Never/1” to “Always/5”:

- When reflecting on your time volunteering in an RiA session: How often do you feel responsible for the safety of the horse?; How often do you feel responsible for the safety of the student?; How often do you feel stressed regarding the safety of the horse?; How often do you feel stressed regarding the safety of the student?

Four questions were short answers:

- In your opinion, what aspects of human-equine interaction are therapeutic?
- Please explain what you liked most about the session you liked the most.
- Do you feel research is important to understand the impact of riding? Why or why not?
- When reflecting on your experience as a volunteer at Fieldstone Farm, why do you volunteer?

### 2.5. Analysis

Analysis of the saliva was conducted by Salimetrics LLC. Oxytocin was assessed using electrochemiluminescence, cortisol was assessed using an enzyme-linked immunosorbent (ELISA) assay, and alpha-amylase was assessed using an enzymatic assay method [15]. The raw data were shared with the research team. The analysis sought to assess the changes pre/post each session in weeks one, four, seven, and ten. This allowed for a picture of the volunteers’ engagement over time. A Wilcoxon signed-rank test was performed to analyze the pre/post changes of each analyte at each time point. A linear mixed-effects model was performed to test the pre/post changes of each analyte across all time points. 

Regarding the volunteer survey, frequencies were analyzed to understand the volunteer’s experience. Short answer questions were analyzed by one researcher of the team. The researcher used phenomenological methodology to understand the participant’s experience of volunteerism and engagement in research [21,22]. The researcher used content analysis because it provides framework for a structural approach that focuses on frequency and patterns of language within responses in addition to presence, meanings, and relationships of certain words, themes, or concept [23]. This process included preparation of the surveys, organizing and coding, and reporting and interpreting [23]. Language (codes) that was repeated through multiple surveys (frequency) was prioritized and considered salient. The salient themes are discussed below. 

## 3. Results

### 3.1. Demographics

The 41 volunteers who consented to the RiA study were consistently serving as horse leaders for the riders. Volunteers in RiA had been volunteers at the Center between 2 (*n* = 5) to 32 (*n* = 2) years, with a mean of 11.39 years of service and median of 10 years of service. Two volunteers identified as male, one as trans/non-binary, and the rest were female. Seventeen were employed, and eighteen were of retirement age. The majority (*n* = 37) lived between 2–25 miles of the Center. Of the 41 volunteers, 19 completed the volunteer survey. 

**Figure 2:**
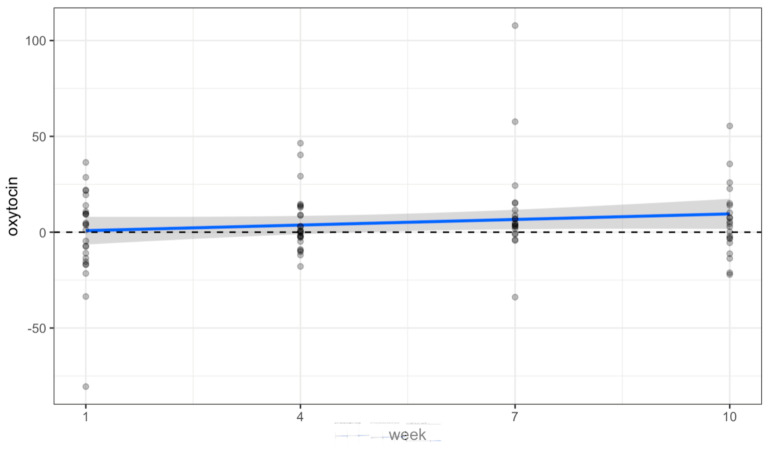
Pre/Post changes of Oxytocin (pg/mL) (*p* = 0.133).

**Figure 3:**
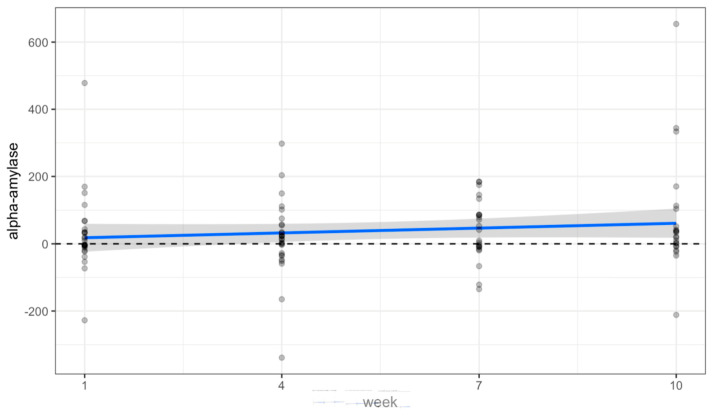
Pre/post changes of Alpha-Amylase (U/mL) (*p* = 0.186).

**Figure 4:**
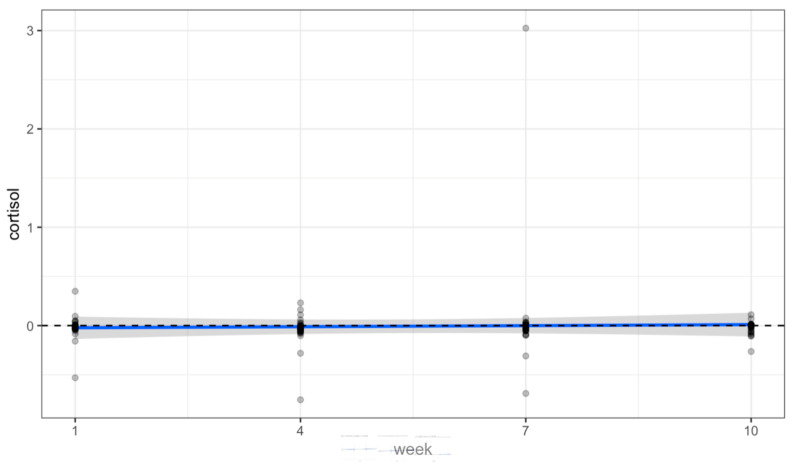
Pre/post changes of Cortisol (µg/dL) (*p* = 0.746).

There were no statistically significant changes in the saliva data collected over time from volunteers. Oxytocin significantly increased at week 7 (*p* = 0.009), but this was an isolated finding not represented at each timepoint (see Table 1). Figure 2, Figure 3 and Figure 4 and Table 1 show a positive trend in oxytocin and alpha-amylase, while cortisol remained level. 

### 3.2. Satisfaction

Of the 41 volunteers who consented, 19 completed the survey (see Table 2). Responses from the volunteers demonstrated that the majority were excited to be a part of research (*n* = 16), did not find it burdensome (*n* = 18), and looked forward to learning about the research findings (*n* = 16). 

Results from the open-ended questions found a shared desire to be present for their rider, to embody a kind yet assertive leader role with their horse, and a sense of responsibility to keep all safe. In addition to giving their time, the volunteers also felt a self-initiated burden of ensuring rider enjoyment and safety while simultaneously enjoying their time. 

A volunteer shared that they “[Felt] like [they were] contributing to better understanding of the benefits of therapeutic riding”. When reflecting on their time in sessions, volunteers felt responsible for the safety of the rider (*n* = 18) and horse (*n* = 19) at least half of the time or more. Many volunteers (*n* = 11) felt stress some of the time to half of the time for the horse, and (*n* = 17) felt stress some to all of the time when thinking of the riders. 

Eighteen respondents shared an affirmative open-ended response that “research is important to understand the impact of riding.” The final question asked volunteers: “When reflecting on your experience as a volunteer at Fieldstone Farm, why do you volunteer?” The responses were positive and illuminated the volunteers’ sense of self within their volunteerism: “The barn is a safe place for ME!”; “The horses and the students, sometimes they do more for me than I do for them…”; “Initially for my love of working with horses. Now, added to that is the impact a horse has on someone’s life.”; and “I volunteer because I know this program makes a difference and it is time for me to be a part of that community”.

## 4. Discussion

The purpose of this study was to explore physiological changes among volunteers in terms of their cortisol and alpha-amylase (stress indicators), and oxytocin (affiliative bonding) levels, and to capture volunteers’ satisfaction with their experience. Several findings are noteworthy. First, many volunteers report experiencing stress and satisfaction simultaneously. Whether the stress is perceived to be positive or negative is open to interpretation and likely to be based on each individual’s experience. Open-ended responses to survey questions suggested that respondents experienced a sense of responsibility for their role and took seriously the need to ensure safety for the riders. They also enjoyed being able to assist the riders, and this contributed to an overall sense of well-being. The competing feelings are consistent with the theoretical frameworks. For example, the psychological contract framework is evidenced by the expressed commitment to the organization by the volunteer. The Social Cure theoretical framework could be used to explain the positive effects from participating in a group, expressed sense of shared identity, and connectedness [4,5,6]. 

Understanding the volunteer’s experience at an individual level, as well as at a collective level, is helpful to ensure the organization can fulfill volunteer expectations. As such, implications of these findings include ensuring the organization provides a reciprocal sense of connectedness for the volunteer to the organization and to the rider(s) they are supporting. Future research may seek to explore the impact of the volunteer on the horse within an EAS session. Doing so may have direct implications on equine-welfare practices within EAS. Bring together research about equine stress [24,25,26] and volunteerism may influence training and practice for humans and horses. Knowing the central and vital role volunteers have in EAS, it may be important to include them in future research on EAS.

Given the cortisol output, it is reasonable to postulate that the volunteers’ experience was not emotionally negative, while the non-significant increase in salivary alpha-amylase may indicate that the stress represented an increase in effort (i.e., physical effort, mental effort). Thus, the volunteers may have experienced stress that was not negative while also experiencing affiliative bonding, evidenced by the non-significant positive trend of oxytocin [16,17]. Taken together, the findings demonstrate the complexity of the human physiological system. Interestingly, the volunteers’ data mirrored that of the horses [13,16] with both stress and affiliative bonding moving in a positive direction. This study supports the physiological concept that humans are biologically complex and are capable of feeling multiple and differing sensations/emotions.

In addition, volunteers expressed high satisfaction with their volunteerism. Again, the volunteer’s sentiment echoes the literature about theoretical frameworks by focusing on commitment, connectedness, safety, and the collective experience [4,5,6]. The volunteers expressed how they found connection in their work with the riders, and for some, with the horses. For several, the opportunity to share a passion that they love, horses and riding, was a meaningful connection. One stated “the research verifies what an intuitive person senses”—while research is needed, the optimism and self-validation of the volunteers is important in understanding their felt sense of commitment.

Understanding the intention of volunteers is important to developing practices to recruit and retain volunteers, which is a necessary component of EAS. While Ben-Porat and colleagues [18] analyzed the negative attributes of volunteerism in this industry, the current study also notes the positive impact of volunteering. The non-significant yet positive trend of oxytocin found in this study may provide initial support for the hypothesis that volunteers enjoy their time and experience a level of affiliative bonding, perhaps with the horse and/or rider. The results of the survey support that time volunteered is an intentional decision to work with the horses and riders as a meaningful act of volunteerism.

### Limitations

The limitations of this study include having a small sample limited by the number of volunteers at the Center during the time of the study. Similarly, the survey portion of the study had a moderate response rate, likely because there was a time gap between the empirical data collection and the survey dissemination. This gap in time was due to the need to obtain an amendment to the original IRB for additional work, and the lengthy amendment process limitations. This study was conducted in a single location, which also is a limitation. The sample size further limits the conclusions that can be drawn.

As referenced above, the study was conducted while COVID-19 precautions were still in place. Volunteers only had the option of leading, and future research could also consider volunteers who sidewalk. Overall, the focus of the full study was the impact of the RiA intervention on youth with anxiety. Inclusion of the volunteers was an intentional aspect of the study design. Further research is suggested to link volunteers’ physiological data with physiological data from riders and horses, as well as the collection of qualitative and quantitative data through mixed-methods research. Ideally, future studies can continue to combine different methods of data collection using physiological measures, self-reports, observational, and survey data, and can use more rigorous design. 

## 5. Conclusions

The 813 Centers in the EAS industry are reliant on volunteers [3]. Yet, volunteers have not been the subjects of research until 2022 [7]. Understanding volunteer effort and impact will help researchers, practitioners, and industry leaders to better understand intentionality of volunteerism and create sustainable opportunities for volunteering. This study builds on the prior peer-reviewed literature by exploring the physiological experience of volunteers pre and post adaptive riding sessions. Continued research about the experiences of volunteers, their impact on adaptive riding, and the EAS industry as a whole, is an important direction for future investigations to deepen knowledge about the impact of adaptive riding on all participants—horses, riders, and those who volunteer. 

Volunteers who lead horses are directly increasing accessibility to a therapeutic sport for persons with disabilities. Furthermore, their service directly supports the bond between horse and rider, and has a direct impact on the horse. They are vital to the safety of the rider and horse throughout the session. The findings demonstrate no significant increase in either stress, as measured by salivary cortisol and amylase, nor in affiliation, as measured by salivary oxytocin. While this responsibility is evidenced by a non-significant increase in stress, it is not necessarily negative stress, as there is evidence that stress can be indicative of effort given to a meaningful task or objective. Simultaneously, affiliative bonding may also be occurring, which may impact negative stress. The same non-significant trends were echoed in both the horses and riders’ experiences [12,13,14] giving additional evidence of the interconnectedness of horse, rider, and volunteer. 

## Figures and Tables

**Table 1:** Means by Analyte.

Week	Analyte	Pre Mean	Post Mean	Difference (Post − Pre)	*p*-Value
Week 1	Oxytocin	31.963	30.629	−1.33	0.895
	Alpha-amylase	158.410	189.288	30.88	0.242
	Cortisol	0.186	0.164	−0.02	0.154
Week 4	Oxytocin	21.587	26.535	4.95	0.287
	Alpha-amylase	143.007	160.124	17.12	0.258
	Cortisol	0.196	0.152	−0.04	0.057
Week 7	Oxytocin	19.137	30.675	11.54	0.009 *
	Alpha-amylase	152.989	194.500	41.51	0.061
	Cortisol	0.187	0.262	0.07	0.121
Week 10	Oxytocin	28.077	34.013	5.94	0.191
	Alpha-amylase	158.512	229.333	70.82	0.035 *
	Cortisol	0.161	0.134	−0.03	0.168

* indicated a significant *p*-value.

**Table 2:** Survey response to: “When thinking about the research process please rate how much you agree with the following statements”.

Survey Question	Agree	Neither Agree nor Disagree	Disagree
I understand why spit was collected	19		
It was burdensome (time) to provide the samples	3	1	15
It was burdensome (process of spitting) to provide the samples	6	2	11

## Data Availability

The supporting information can be accessed by emailing the corresponding author and at www.reininginanxiety.com.

## References

[1] Census National Volunteer Week. https://www.census.gov/newsroom/stories/volunteer-week.html.

[2] Independent Sector Value of Time. https://independentsector.org/resource/value-of-volunteer-time/.

[3] PATH Intl 2020 Fact Sheet. https://pathintl.org/wp-content/uploads/2022/03/PATH-facts-2022.pdf.

[4] Bailey E.L., Waite K., Wilson K.M. (2013). HALTER: Using HorseQuest as a Training Tool. J. Ext..

[5] Culp K., Bullock L.R. (2017). 4-H Volunteer Continuing Education Academy. J. Ext..

[6] Gray D., Stevenson C. (2020). How can ‘we’ help? Exploring the role of shared social identity in the experiences and benefits of volunteering. J. Community Appl. Soc. Psychol..

[7] Haski-Leventhal D., Paull M., Young S., MacCallum J., Holmes K., Omari M., Scott R., Alony I. (2020). The multidimensional benefits of university student volunteering: Psychological contract, expectations, and outcomes. Nonprofit Volunt. Sect. Q..

[8] Jongenelis M.I., Dana L.M., Warburton J., Jackson B., Newton R.U., Talati Z., Pettigrew S. (2020). Factors associated with formal volunteering among retirees. Eur. J. Ageing.

[9] Rudd C., Wheeler B., Pasiuk E., Schroeder K. (2022). An Initial Survey of Volunteer Perceptions of Horses in Equine-Assisted Services: Volunteer Experiences, Training, and Educational Needs. J. Equine Vet. Sci..

[10] St Peter C.C., Shuler N.J., Jones S.H., Bradtke S., Hull S.L., Browning B., VanGilder S., Petitto C. (2021). Comparing training methods to improve volunteer skills during therapeutic horseback riding: A randomized control trial. J. Appl. Behav. Anal..

[11] Hoye R., Kappelides P. (2021). The psychological contract and volunteering: A systematic review. Nonprofit Manag. Leadersh..

[12] Acri M., Morrissey M., Peth-Pierce R., Seibel L., Seag D., Hamovitch E.K., Guo F., Horwitz S., Hoagwood K.E. (2021). An equine-assisted therapy for youth with mild to moderate anxiety: Manual development and fidelity. J. Child. Fam. Stud..

[13] Hoagwood K.E., Acri M., Morrissey M., Peth-Pierce R., Seibel L., Seag D.E., Vincent A., Guo F., Hamovitch E.K., Horwitz S. (2021). Adaptive Riding Incorporating Cognitive-Behavioral Elements for Youth with Anxiety: An Exploratory Randomized Controlled Study. Hum. Anim. Interact. Bull..

[14] Hoagwood K., Vincent A., Acri M., Morrissey M., Seibel L., Guo F., Flores C., Seag D., Peth Pierce R., Horwitz S. (2022). Reducing Anxiety and Stress among Youth in a CBT-Based Equine-Assisted Adaptive Riding Program. Animals.

[15] Wu Y., Veerareddy A., Lee M.R., Bellucci G., Camilleri J.A., Eickhoff S.B., Krueger F. (2021). Understanding identification-based trust in the light of affiliative bonding: Meta-analytic neuroimaging evidence. Neurosci. Biobehav. Rev..

[16] Vincent A., Peth-Pierce R.M., Morrissey M.A., Acri M.C., Guo F., Seibel L., Hoagwood K.E. (2021). Evaluation of a Modified Bit Device to Obtain Saliva Samples from Horses. Vet. Sci..

[17] Salimetrics LLC Analytes in Saliva Quick Reference Guide. https://salimetrics.com/all-analytes-dna/.

[18] Aschbacher K., O’Donovan A., Wolkowitz O.M., Dhabhar F.S., Su Y., Epel E. (2013). Good stress, bad stress and oxidative stress: Insights from anticipatory cortisol reactivity. Psychoneuroendocrinology.

[19] Gordis E.B., Granger D.A., Susman E.J., Trickett P.K. (2006). Asymmetry between salivary cortisol and α-amylase reactivity to stress: Relation to aggressive behavior in adolescents. Psychoneuroendocrinology.

[20] Ben-Porat A., Gil L., Brafman D., Zriker A., Levy D. (2021). Secondary traumatic stress and posttraumatic growth among volunteers at a therapeutic riding center: The role of personal and environmental factors. Curr. Psychol..

[21] Neubauer B.E., Witkop C.T., Varpio L. (2019). How phenomenology can help us learn from the experiences of others. Persp. Med. Educ..

[22] Alhazmi A.A., Kaufmann A. (2022). Phenomenological qualitative methods applied to the analysis of cross-cultural experience in novel educational social contexts. Front. Psychol..

[23] Atlas.ti The Ultimate Guide to Qualitative Research—Part 2: Handling Qualitative Data. https://atlasti.com/guides/qualitative-research-guide-part-2/content-analysis-vs-thematic-analysis.

[24] McKinney C., Mueller M.K., Frank N. (2015). Effects of therapeutic riding on measures of stress in horses. J. Equine Vet. Sci..

[25] Ferlazzo A., Fazio E., Cravana C., Medica P. (2023). Equine-assisted services: An overview of current scientific contributions on efficacy and outcomes on humans and horses. J. Vet. Behav..

[26] Rankins E.M., McKeever K.H., Malinowski K. (2003). Equids in Equine Assisted Services: A Scoping Review. J. Equine Vet. Sci..

